# Hypoxic glioma-derived exosomes promote M2-like macrophage polarization by enhancing autophagy induction

**DOI:** 10.1038/s41419-021-03664-1

**Published:** 2021-04-07

**Authors:** Jianye Xu, Jian Zhang, Zongpu Zhang, Zijie Gao, Yanhua Qi, Wei Qiu, Ziwen Pan, Qindong Guo, Boyan Li, Shulin Zhao, Xiaofan Guo, Mingyu Qian, Zihang Chen, Shaobo Wang, Xiao Gao, Shouji Zhang, Huizhi Wang, Xing Guo, Ping Zhang, Rongrong Zhao, Hao Xue, Gang Li

**Affiliations:** 1 Department of Neurosurgery, Qilu Hospital, Cheeloo College of Medicine and Institute of Brain and Brain-Inspired Science, Shandong University, Jinan, 250012 Shandong China; 2 Shandong Key Laboratory of Brain Function Remodeling, Jinan, 250012 Shandong China; 3 Department of Neurosurgery, Dezhou People’s Hospital, Dezhou, 253000 Shandong China

**Keywords:** Cancer microenvironment, Autophagy

## Abstract

Exosomes participate in intercellular communication and glioma microenvironment modulation, but the exact mechanisms by which glioma-derived exosomes (GDEs) promote the generation of the immunosuppressive microenvironment are still unclear. Here, we investigated the effects of GDEs on autophagy, the polarization of tumor-associated macrophages (TAMs), and glioma progression. Compared with normoxic glioma-derived exosomes (N-GDEs), hypoxic glioma-derived exosomes (H-GDEs) markedly facilitated autophagy and M2-like macrophage polarization, which subsequently promoted glioma proliferation and migration in vitro and in vivo. Western blot and qRT-PCR analyses indicated that interleukin 6 (IL-6) and miR-155-3p were highly expressed in H-GDEs. Further experiments showed that IL-6 and miR-155-3p induced M2-like macrophage polarization via the IL-6-pSTAT3-miR-155-3p-autophagy-pSTAT3 positive feedback loop, which promotes glioma progression. Our study clarifies a mechanism by which hypoxia and glioma influence autophagy and M2-like macrophage polarization via exosomes, which could advance the formation of the immunosuppressive microenvironment. Our findings suggest that IL-6 and miR-155-3p may be novel biomarkers for diagnosing glioma and that treatments targeting autophagy and the STAT3 pathway may contribute to antitumor immunotherapy.

## Introduction

Human glioma, the most common and lethal primary intracranial tumor, displays an aggressive malignant progression characterized by the devastation to normal brain tissue, widespread invasion throughout the brain, and resistance to therapeutic approaches^1,2^. Despite optimal treatment, glioblastoma (GBM, WHO grade IV) patients still have a median survival of 12–15 months^3,4^. The unique immunosuppressive tumor microenvironment of malignant glioma consists of many diverse factors and varied cell types, such as tumor cells, fibroblasts, and multiple types of immune cells, and promotes resistance to multiple therapies^5^.

Hypoxia is one characteristic of the glioma microenvironment^6,7^. Moreover, macrophages, the primary immune cells residing in the glioma microenvironment, preferentially accumulate in hypoxic areas, where they can polarize into specific cell types^8,9^. Two types of polarized macrophage phenotypes have been identified: classically activated macrophages (M1-like type) and alternatively activated macrophages (M2-like type)^10^. It has been reported that M2-like type macrophages drastically facilitate glioma progression and are associated with a poor prognosis of glioma patients^11^. Tumor-associated macrophages (TAMs), the most abundantly infiltrating immune cells in the glioma microenvironment, are more likely to become M2-like type macrophages in the immunosuppressive glioma microenvironment^12^. In addition, M2-like TAMs play an important role in tumor progression, promoting an immunosuppressive signal in the tumor^13^. Furthermore, our previous study reported that hypoxia can promote M2-like type macrophage polarization^14^.

Exosomes are small membrane vesicles (30–100 nm in size) that can be secreted by several types of cells^15^. As an important component of the tumor microenvironment, exosomes, which contain microRNAs (miRNAs) and proteins, play crucial roles in intercellular communication by delivering their content^16,17^. Tumor exosomes have been affirmed to transfer genetic materials and suppress immune cell function^18^. Interestingly, recent studies have reported that hypoxia can alter the content and enhance the release of exosomes, thereby influencing recipient cell functions by regulating cell-cell communication^19–21^. Our team has demonstrated that hypoxia can induce the immunosuppressive function of myeloid-derived suppressor cells (MDSCs) by regulating the content of glioma-derived exosomes (GDEs)^22,23^.

Autophagy/macroautophagy, which means “self-eating”, is a process that involves the formation of double-membrane vesicles (autophagosomes) that engulf cytoplasmic structures and fuse with lysosomes for degradation^24^. Autophagy provides cells with essential elements for survival during hypoxia, starvation, immune responses, and chemoradiotherapy^25^. The dysregulation of autophagy plays an important role in human tumorigenesis^25,26^.

The interleukin (IL)-6-signal transducer and activator of transcription 3 (STAT3) pathway is hyperactivated in many types of cancers and is involved in tumor progression^27^, and miR-155-3p is overexpressed and promotes tumorigenesis in various cancers^28–30^. Our team has reported that hypoxia-induced IL-6 and miR-155-3p serve as significant autophagy initiators through STAT3 and cyclic adenosine monophosphate (CAMP) responsive element-binding protein 3 (CREB3) activation in GBM^31^. In addition, autophagy has been reported to induce M2-like macrophage polarization via activation of STAT3 pathway^32,33^. Therefore, the STAT3 pathway may be a central pathway of IL-6 and miR-155-3p that promotes M2-like macrophage polarization by enhancing the induction of autophagy.

In this study, we investigated whether hypoxic glioma-derived exosomes (H-GDEs) induced autophagy in TAMs and promoted M2-like macrophage polarization. We first demonstrated that H-GDEs could induce M2-like macrophage polarization via autophagy activation. According to our previous studies, hypoxia induces the upregulation of IL-6 and miR-155-3p in GBM cells^31^. Then, we detected and compared the content of IL-6 and miR-155-3p between H-GDEs and normoxic glioma-derived exosomes (N-GDEs) and found that the expression levels of IL-6 and miR-155-3p were higher in H-GDEs than in N-GDEs. Furthermore, we investigated the function of STAT3 pathway activation in the IL-6-autophagy-M2-like macrophage polarization process using S3I-201, a STAT3 inhibitor. Finally, we showed that IL-6 and miR-155-3p, which were delivered by exosomes, enhanced autophagy induction and M2-like macrophage polarization, creating a positive feedback loop via activation of the STAT3 pathway.

## Materials and methods

### Cell culture

The human GBM cell lines U87MG and U251 and the human monocyte cell lines U937 and THP-1 were obtained from the Chinese Academy of Sciences Cell Bank. GBM cell lines were cultured in Dulbecco’s modified Eagle’s medium (DMEM, Thermo Fisher Scientific, USA) supplemented with 10% fetal bovine serum (FBS, Thermo Fisher Scientific). U937 and THP-1 cells were cultured in RPMI-1640 (Thermo Fisher Scientific) supplemented with 10% FBS (Thermo Fisher Scientific). U937 and THP-1 cells were incubated with 100 ng/ml phorbol 12-myristate 13-acetate (PMA, Sigma-Aldrich, USA) for 24 h to induce their differentiation into macrophages in vitro. For exosome coculture, 1 μg/ml exosomes were added to the culture medium of recipient cells, as previously reported^7,23,34^. To investigate the role of macrophages in the progression of glioma, an in vitro coculture system was used: macrophages cultured in 6-well plates were treated with exosomes or transfected; after 48 h, glioma cells were cultured in the upper chamber.

### Exosome isolation

GBM cell lines were cultured in DMEM supplemented with 10% exosome-depleted FBS under normoxic (21% O_2_) or hypoxic (1% O_2_) conditions. Exosomes were isolated from the cell culture supernatant using several centrifugation and ultracentrifugation steps as previously described^35^. The GDEs were stored at −80 °C.

### Electron microscopy

Isolated exosomes were loaded onto a carbon-coated electron microscopy grid and examined using transmission electron microscopy (TEM). One drop of glutaraldehyde (3%) was placed on the grids and incubated for 5 min. Then, the grids were washed with three-fold-distilled water for 2 min for a total of ten washes. Next, the grids were processed with uranyl-acetate solution (4%) for 10 min and methylcellulose solution (1%) for 5 min to compare the exosome samples. Grids were dried and observed using a TEM-1011 electron microscope at 80 kV (JEOL-1200EX, Japan).

### Western blot

Protein was extracted from GDEs and macrophages. Protein lysates were loaded and separated on SDS-PAGE, and the proteins were transferred to polyvinylidene difluoride (PVDF) membranes. The blots were incubated with primary antibodies against CD9 (System Biosciences, USA), TSG101, CREBRF, CREB3, ATG5, LC3B, IL-6, STAT3 (Abcam, UK), Calnexin, p-STAT3 (Y705), P62, and GAPDH (Cell Signaling Technology, USA).

### Virus transfection, inhibitors of autophagy, and STAT3 activation

The IL-6 and miR-155-3p overexpression and control lentiviruses were synthesized by GeneChem (Shanghai, China). Autophagy was inhibited by 3-methyladenine (3-MA, 500 μmol/L, MedChemExpress, USA). STAT3 activation was inhibited by S3I‐201 (25 μmol/L, NSC 74859, MedChemExpress).

### RNA extraction and qRT-PCR

Exosome RNA extraction was conducted using the SeraMirTM Exosome RNA Extraction Kit (System Biosciences, USA) after isolation of exosomes. TRIzol was used to extract total cell RNA according to the manufacturer’s protocol. Reverse transcription was performed using High Capacity cDNA Reverse Transcription Kits (Applied Bio-systems) according to the manufacturer’s protocols. The cDNA was subjected to real-time PCR using the quantitative PCR System Mx-3000P (Stratagene). The PCR primer sequences were as follows: GAPDH: forward, 5′-GCACCGTCAAGGCTGAGAAC-3′ and reverse, 5′-TGGTGAAGACGCCAGTGGA-3′; CD163: forward, 5′-GGCTTGCAGTTTCCTCAAGA-3′ and reverse, 5′-GACACAGAAATTAGTTCAGCAGCA-3′; TNFA: forward, 5′-CTGCACTTTGGAGTGATCGG-3′ and reverse, 5′-TCAGCTTGAGGGTTTGCTAC-3′; CREBRF: forward, 5′-TGAACTGGATAGAGAGATGAACTAC-3′ and reverse, 5′-CCACTGTTCCCAGTTTGAGGT-3′; U6: forward, 5′-ATTGGAACGATACAGAGAAGATT-3′ and reverse, 5′-GGAACGCTTCACGAATTTG-3′; and miR-155-3p: forward, 5′-GGCGAATCTCCTACATATTAGCA-3′ and reverse, 5′-TATGGTTTTGACGACTGTGTGAT-3′.

### Lentivirus-mCherry-GFP-LC3 transfection and confocal microscopy

U937 and THP-1 cells were cultured in 24-well plates and transfected with lentiviruses expressing the mCherry-GFP-LC3 plasmid. The cells stably expressing mCherry-GFP-LC3 were incubated with PMA to induce their differentiation into macrophages. Subsequently, the cells were treated with various agents. Then, the images of mCherry-GFP-LC3-macrophages were visualized with a Leica TCS SP8 confocal microscope.

### Flow cytometry

To detect CD11b + CD163+ macrophages, anti-CD163-PE (BD Biosciences, USA) and anti-CD11b-APC (eBioscience, USA) were used to stain cells. Isotype controls were run in parallel. Flow cytometry was performed using a BD Accuri C6 flow cytometer (BD Biosciences).

### Cytokine assay

Cell culture medium was collected 72 h after the indicated treatment. The secretion of IL-10 was detected by enzyme-linked immunosorbent assay (ELISA; Proteintech, USA) according to the manufacturer’s instructions.

### 5-Ethynyl-2′-deoxyuridine (EdU) cell proliferation assay

Cell proliferation rates were measured by an EdU cell proliferation assay kit (RiboBio, #C10310-1; China). Cells were incubated with 200 μL of 5-ethynyl-20-deoxyuridine (EdU) for 2 h at 37 °C. Cells were fixed in 4% paraformaldehyde for 20 min, permeabilized with 0.4% Triton X-100 for 10 min, and incubated with Apollo® reagent (100 μL) for 30 min. The cells were stained with Hoechst for 30 min, and representative images were obtained using a Leica-inverted fluorescence microscope. The cell proliferation rate was assessed using the ratio of EdU-positive cells (red) to total Hoechst-positive cells (blue).

### Transwell assay

Glioma cells were added to the top chamber in serum-free media. The bottom chamber was filled with 10% FBS DMEM. After 24–48 h of incubation, the top chamber cells were removed using a cotton swab, and the membrane was fixed in 4% paraformaldehyde for 15 min and stained with crystal violet for 15 min.

### Luciferase reporter assays

Macrophages were cotransfected with firefly luciferase reporters and the indicated plasmids using Lipofectamine 3000 (Invitrogen/Thermo Fisher Scientific), and luciferase assays were performed 24 h later using the Dual-Luciferase Reporter Assay Kit (Promega). Renilla activity was used to normalize the luciferase reporter activity. The reporter genes containing GV272-CREBRF-3′UTR and GV272-mut-CREBRF-3’UTR were synthesized by Genechem (Shanghai, China).

### Intracranial mouse model

To investigate the roles of H-GDEs in vivo, tumor xenografts were established with 4-week-old male BALB/c nude mice purchased from the Model Animal Research Center of Nanjing University (Nanjing, China). Macrophages were treated with phosphate-buffered saline (PBS), N-GDEs, or H-GDEs for 48 h before implantation. Then, luciferase-labeled U87MG cells (1,000,000 cells per mouse) mixed with different treated macrophages (200,000 cells per mouse) were stereotactically implanted into the brains of the nude mice. After implantation, intravenous injection of PBS, N-GDEs, or H-GDEs in the three groups of mice was performed every three days. The tumor volume was measured and quantified after implantation by in vivo bioluminescent imaging using IVIS Lumina Series III (PerkinElmer, USA). Next, we randomly chose three mice in each group and euthanized them on the same day (20 days after implantation). The brains were fixed with paraformaldehyde for hematoxylin-eosin (HE) and immunohistochemistry (IHC) staining. The remaining mice were kept until death for survival analysis.

For the functional in vivo experiments of IL-6 and miR-155-3p, U937 cells were transfected with lenti-ov-IL-6, lenti-control, lenti-ov-miR-155-3p or lenti-miR-control virus and incubated with PMA (100 ng/ml) for 24 h in vitro to induce them to differentiate into macrophages. Luciferase-labeled U87MG cells (1,000,000 cells per mouse) were mixed with 200,000 conditioned macrophages and injected into the brains of nude mice. Bioluminescence imaging was used to image the tumors at 5 and 15 days after implantation. Next, we randomly chose three mice in each group and euthanized them 20 days after implantation. The brains were fixed with paraformaldehyde for HE and IHC staining. The remaining mice were kept until death for survival analysis.

All procedures that involved animals were approved by the Animal Care and Use Committee of Qilu Hospital of Shandong University.

### Immunohistochemistry

IHC staining was performed as previously described^36^. Briefly, sections were obtained from formalin-fixed, paraffin-embedded tissues of different xenograft models. The slides were blocked with 10% normal goat serum and incubated with primary antibodies (anti-Ki-67 antibody, Cell Signaling Technology, USA) at 4 °C overnight. The signal was visualized using standard protocols, with horseradish-peroxidase-conjugated secondary antibodies and 3,3′-diaminobenzidine (DAB) as the substrate. Then, the slides were counterstained with hematoxylin, and typical images were obtained using a Leica DM 2500 microscope.

### Statistical analysis

A one-way ANOVA test or Student’s *t* test was used for all other data comparisons using Statistical Product and Service Solutions (SPSS) software. All data are presented as the means ± SD. All tests were two-sided, and *P*-values < 0.05 were considered significant. Data visualization was performed using GraphPad Prism 6.

## Results

### Identification of GDEs

We isolated and characterized exosomes derived from the supernatant of U87MG, and U251 glioma cells. TEM images showed that the GDEs were rounded particles ranging from 30 to 100 nm in diameter (Fig. S1a). The exosomes expressed typical exosomal markers, including TSG101 and CD9, and did not express the exosomal negative marker calnexin (Fig. S1b). Human U937 and THP-1 monocytes were treated with PMA to induce their differentiation into macrophages. To confirm the phagocytosis of GDEs by macrophages, macrophages were cocultured with PKH67-labeled GDEs. PKH67 signals were detected in the macrophages, indicating their efficient uptake of GDEs (Fig. S1c).

### H-GDEs enhance autophagy in macrophages

**Fig. 1:**
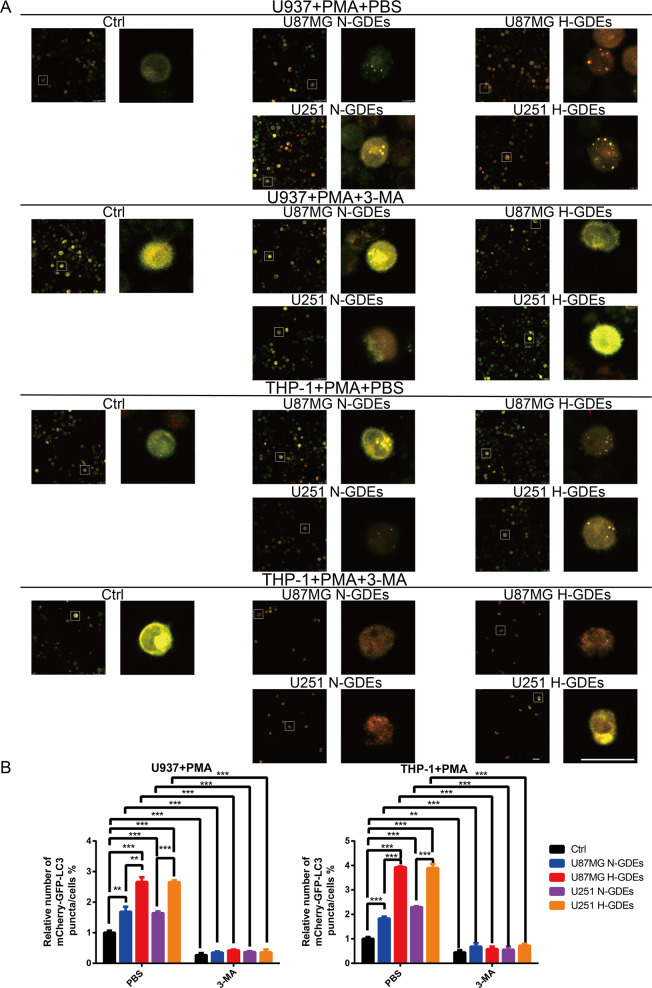
H-GDEs significantly induce autophagy in macrophages in vitro. **A** Human monocyte cell lines U937 and THP-1 were transfected with lentiviruses expressing mCherry‐GFP‐LC3 plasmid and incubated with PMA (100 ng/ml) for 24 h in vitro to induce them to differentiate into macrophages. Then, the macrophages were treated with PBS, N-GDEs, or H-GDEs isolated from the culture supernatants of U87MG or U251 cells. 3-MA was used to inhibit autophagy. Macrophages of different groups were visualized by confocal microscopy. Representative images are shown (Scale bar, 25 μm). **B** The number of mCherry‐GFP-LC3 puncta in each cell was quantified. (*P < 0.05; **P < 0.01; ***P < 0.001; *n* = 3).

To explore the effects of H-GDEs on autophagy in macrophages, U937 and THP-1 cells were transfected with mCherry-GFP-LC3 and incubated with PMA to induce their differentiation into macrophages. After 1 day, the mCherry-GFP-LC3-macrophages were treated with PBS, N-GDEs or H-GDEs, and 3-MA was used to inhibit autophagy. Changes in the puncta/cell ratio were analyzed by confocal microscopy. Compared with PBS and N-GDEs, H-GDEs significantly increased the puncta/cell ratio in macrophages (Fig. 1A, B). In addition, the puncta/cell ratio decreased in macrophages treated with 3-MA, an inhibitor of autophagy (Fig. 1A, B). Next, we investigated the expression of ATG5, P62 and LC3B in macrophages treated with PBS, N-GDEs or H-GDEs by western blot analysis. Consistent with the change in the puncta to cell ratio, H-GDEs significantly promoted ATG5 and LC3B expression and inhibited P62 expression (Fig. S2a). Moreover, 3-MA inhibited the function of H-GDEs (Fig. S2a). Collectively, these results demonstrate that H-GDEs enhance autophagy in macrophages, which can be inhibited by 3-MA.

### H-GDEs promote M2-like macrophage polarization and can be inhibited by 3-MA

**Fig. 2:**
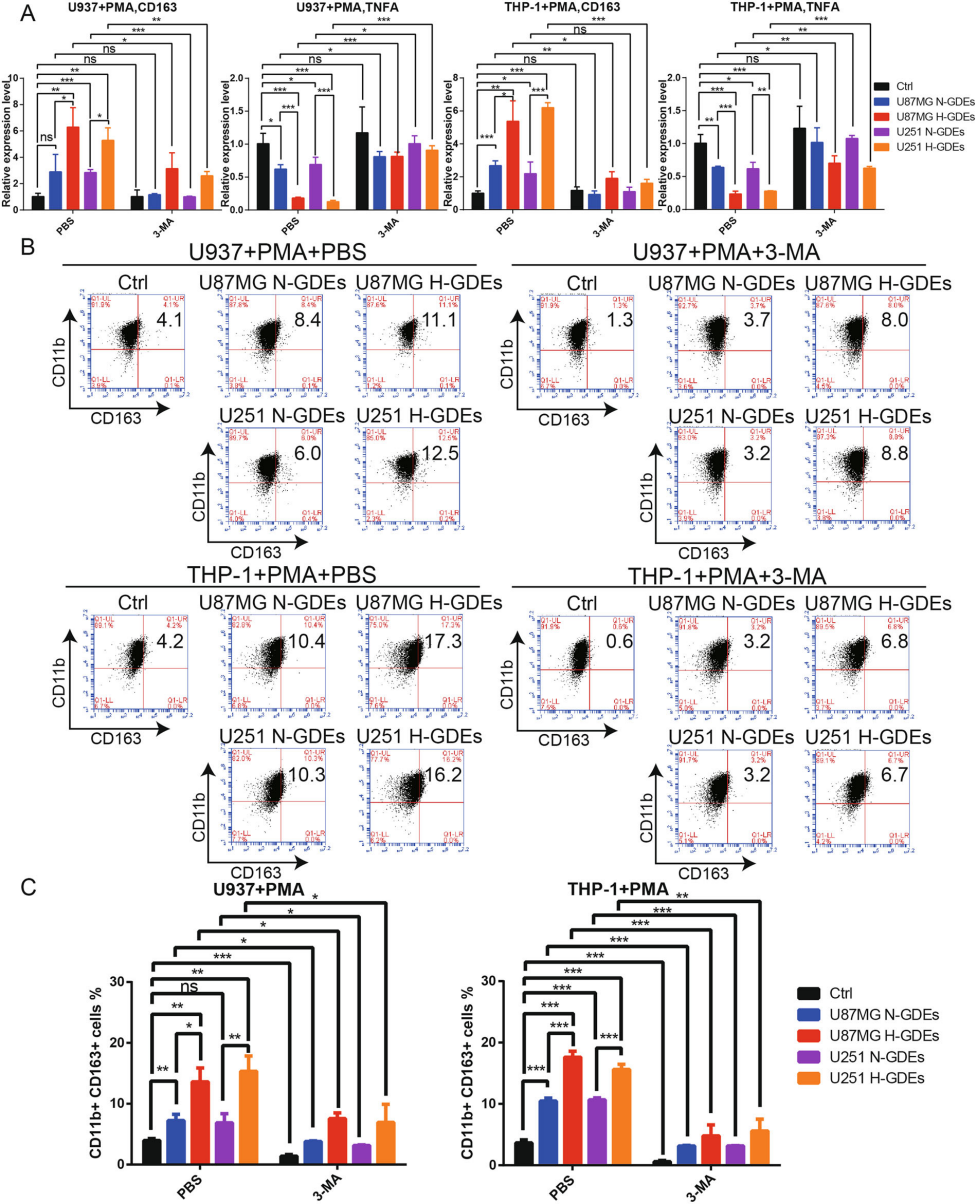
H-GDEs significantly induce M2-like macrophage polarization and can be inhibited by 3-MA. **A** Human monocyte cell lines U937 and THP-1 were incubated with PMA (100 ng/ml) for 24 h in vitro to induce them to differentiate into macrophages. Then, the macrophages were treated with PBS, N-GDEs or H-GDEs isolated from the culture supernatants of U87MG and U251 cells. 3-MA was used to inhibit autophagy. The expression levels of CD163 and TNFA were determined by qRT-PCR. **B**, **C** Macrophages were treated in the same way as described in (**A**). Flow cytometry and quantification were performed to analyze the proportion of CD11b + CD163 + macrophages. (*P < 0.05; **P < 0.01; ***P < 0.001; *n* = 3).

To determine whether H-GDEs could promote M2-like macrophage polarization, macrophages were treated with PBS, N-GDEs or H-GDEs. Quantitative real-time PCR (qRT-PCR) was performed to validate the expression of CD163 and TNFA, which are M2- and M1-like macrophage markers, respectively^37^. Compared with PBS and N-GDEs, H-GDEs significantly increased CD163 expression and markedly decreased TNFA expression (Fig. 2A). Next, we investigated CD163 expression in macrophages by flow cytometry. Interestingly, H-GDEs significantly promoted CD163 expression (Fig. 2B, C). Furthermore, we measured the secretion of IL-10 in macrophage culture supernatants by ELISA. Compared with PBS and N-GDEs, H-GDEs significantly increased the secretion of IL-10 in macrophages (Fig. S2b). Taken together, these results indicated that H-GDEs could induce M2-like macrophage polarization. To validate the correlation between autophagy and M2-like macrophage polarization, we added 3-MA to macrophages treated with PBS, N-GDEs or H-GDEs. 3-MA inhibited the expression of CD163 and the secretion of IL-10 and promoted TNFA expression, which demonstrated that autophagy in macrophages promoted M2-like macrophage polarization (Fig. 2A–C, Fig. S2b).

### Macrophages treated with H-GDEs promote glioma progression in vitro and in vivo

**Fig. 3:**
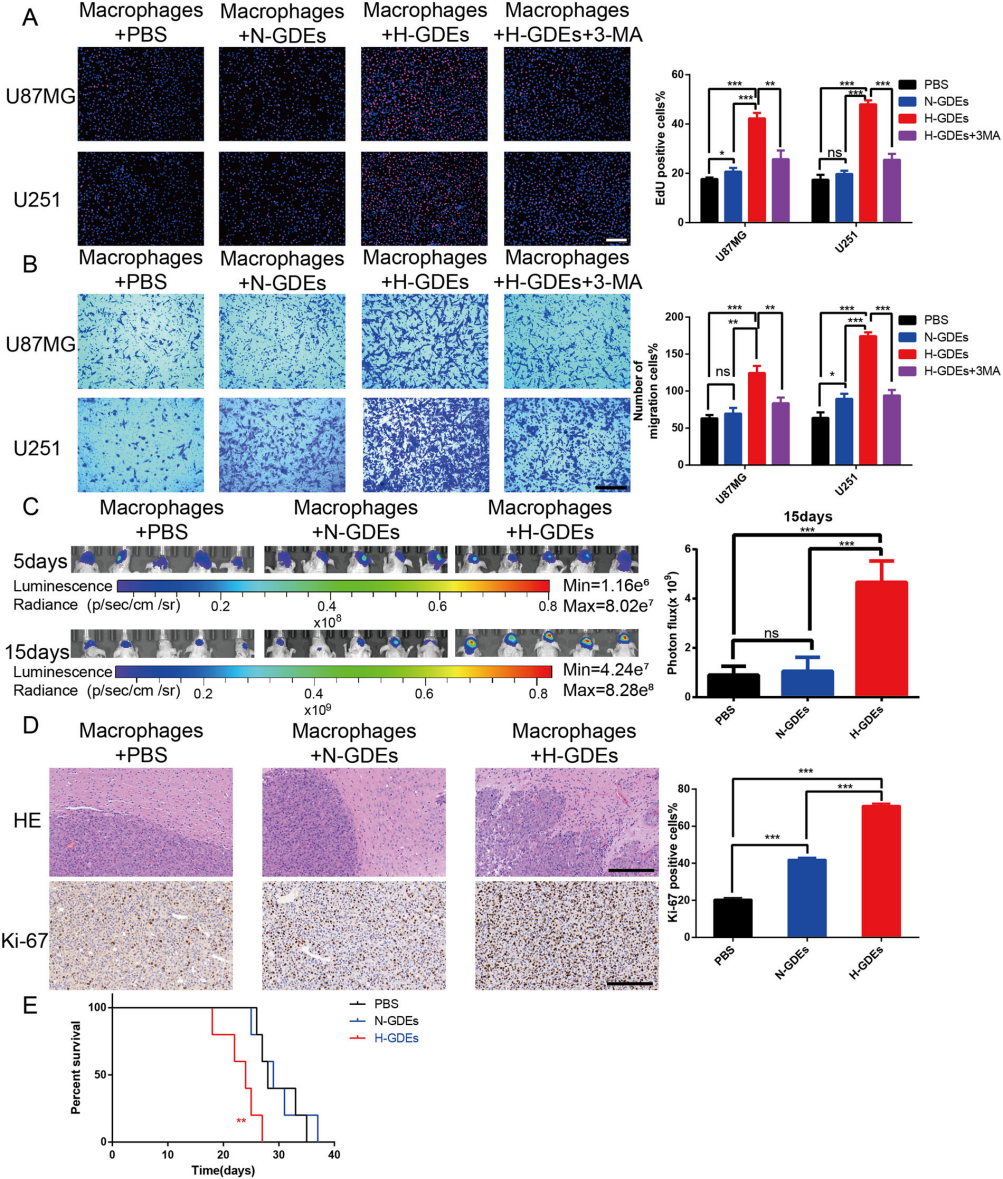
Macrophages treated with H-GDEs promote glioma progression in vitro and vivo. **A** Human monocyte U937 cells were incubated with PMA (100 ng/ml) for 24 h in vitro to induce them to differentiate into macrophages. An EdU assay evaluated the proliferation of U87MG or U251 cells cocultured with macrophages treated with PBS, N-GDEs, H-GDEs, or H-GDEs+3-MA, and the results were quantified (scale bar, 100 μm). **B** The migration capacity of U87MG or U251 cells cocultured with conditioned macrophages was determined. Representative images of migratory cells and quantifications are shown (scale bar, 200 μm). **C** In vivo bioluminescent imaging analysis of tumor growth in xenograft nude mice bearing U87MG cells with PBS-macrophages, N-GDEs-macrophages, or H-GDEs-macrophages. Representative images on day 5 and 15 post-implantation are shown (data are from five mice per group). **D** HE staining and IHC staining for Ki-67 of sections from xenograft mouse brains with U87MG and PBS-macrophages, U87MG and N-GDE-macrophages or U87MG and H-GDE-macrophages on the day of euthanasia (scale bar, 200 μm). **E** Survival analysis of animals implanted with U87MG and PBS-macrophages, U87MG and N-GDEs-macrophages or U87MG and H-GDEs-macrophages (*P* < 0.01 by log-rank analysis; data from five animals per group). (*P < 0.05; **P < 0.01; ***P < 0.001).

To investigate the effects of macrophages treated with GDEs on the progression of glioma, a coculture of macrophages and glioma cells was performed. EdU and transwell assays showed that macrophages treated with H-GDEs significantly promoted glioma cell proliferation and migration, which could be inhibited by 3-MA (Fig. 3A, B). To further examine the effect of H-GDEs on M2-like macrophage polarization in vivo, macrophages treated with PBS, N-GDEs, or H-GDEs were co-implanted with glioma cells into the brains of nude mice. Subsequently, PBS, N-GDEs, or H-GDEs were injected every three days. Fifteen days after implantation, the tumor sizes were examined by bioluminescence imaging, and tumors were enlarged in animals from the H-GDE group (Fig. 3C). HE and IHC staining showed that macrophages treated with H-GDEs had less defined borders and higher expression of Ki-67 (Fig. 3D). Moreover, the survival rate of animals implanted with H-GDE macrophages and glioma was shorter than that of the other groups (Fig. 3E).

### IL-6 and miR-155-3p are highly expressed in H-GDEs and are delivered to macrophages via exosomes

**Fig. 4:**
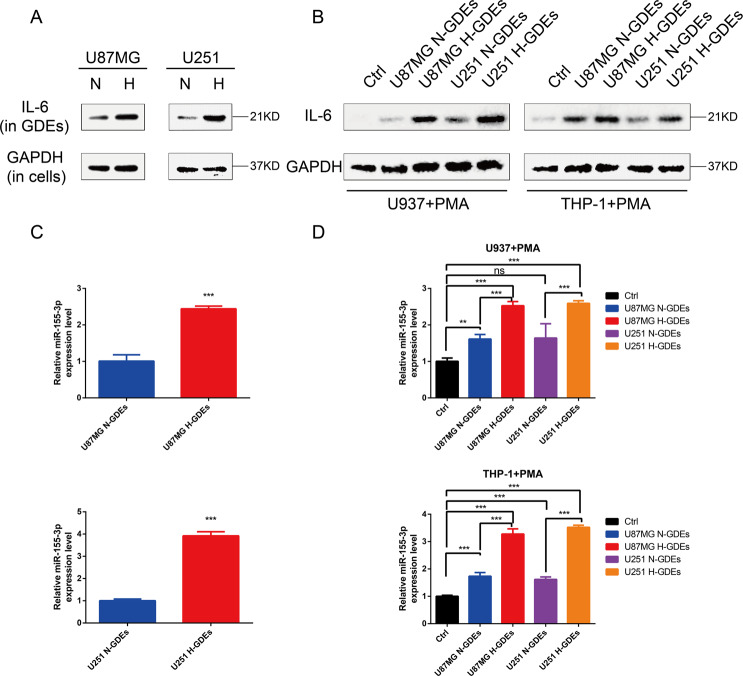
IL-6 and miR-155-3p are highly expressed in H-GDEs and delivered to macrophages via exosomes. **A** The expression levels of IL-6 in normoxic and hypoxic exosomes of U87MG and U251 glioma cell lines were determined by western blot. **B** The expression levels of IL-6 in macrophages treated with PBS, N-GDEs or H-GDEs were determined by western blot. **C** The expression levels of miR-155-3p in normoxic and hypoxic exosomes of U87MG and U251 glioma cell lines were determined by qRT-PCR. **D** The expression levels of miR-155-3p in macrophages treated with PBS, N-GDEs or H-GDEs were determined by qRT-PCR.

Our team previously reported that hypoxia treatment induces a significant increase in IL-6 secretion in glioma cell culture supernatants^31^. To investigate whether the secretion of IL-6 in H-GDEs is higher than that in N-GDEs, we detected IL-6 expression in H-GDEs and N-GDEs by western blotting. IL-6 expression was significantly increased in H-GDEs (Fig. 4A). Moreover, macrophages treated with H-GDEs had higher IL-6 expression than those treated with PBS or N-GDEs (Fig. 4B). In addition, we found that the levels of miR-155-3p in hypoxic glioma cells were increased^31^. To further investigate whether miR-155-3p expression is increased in H-GDEs, qRT-PCR was performed. We found that miR-155-3p expression in H-GDEs was significantly higher than that in N-GDEs (Fig. 4C). Moreover, macrophages incubated with H-GDEs had higher expression levels of IL-6 and miR-155-3p than those incubated with PBS or N-GDEs (Fig. 4D). These findings indicated that IL-6 and miR-155-3p are delivered to macrophages via GDEs.

### IL-6 enhances autophagy induction via STAT3 activation in macrophages

**Fig. 5:**
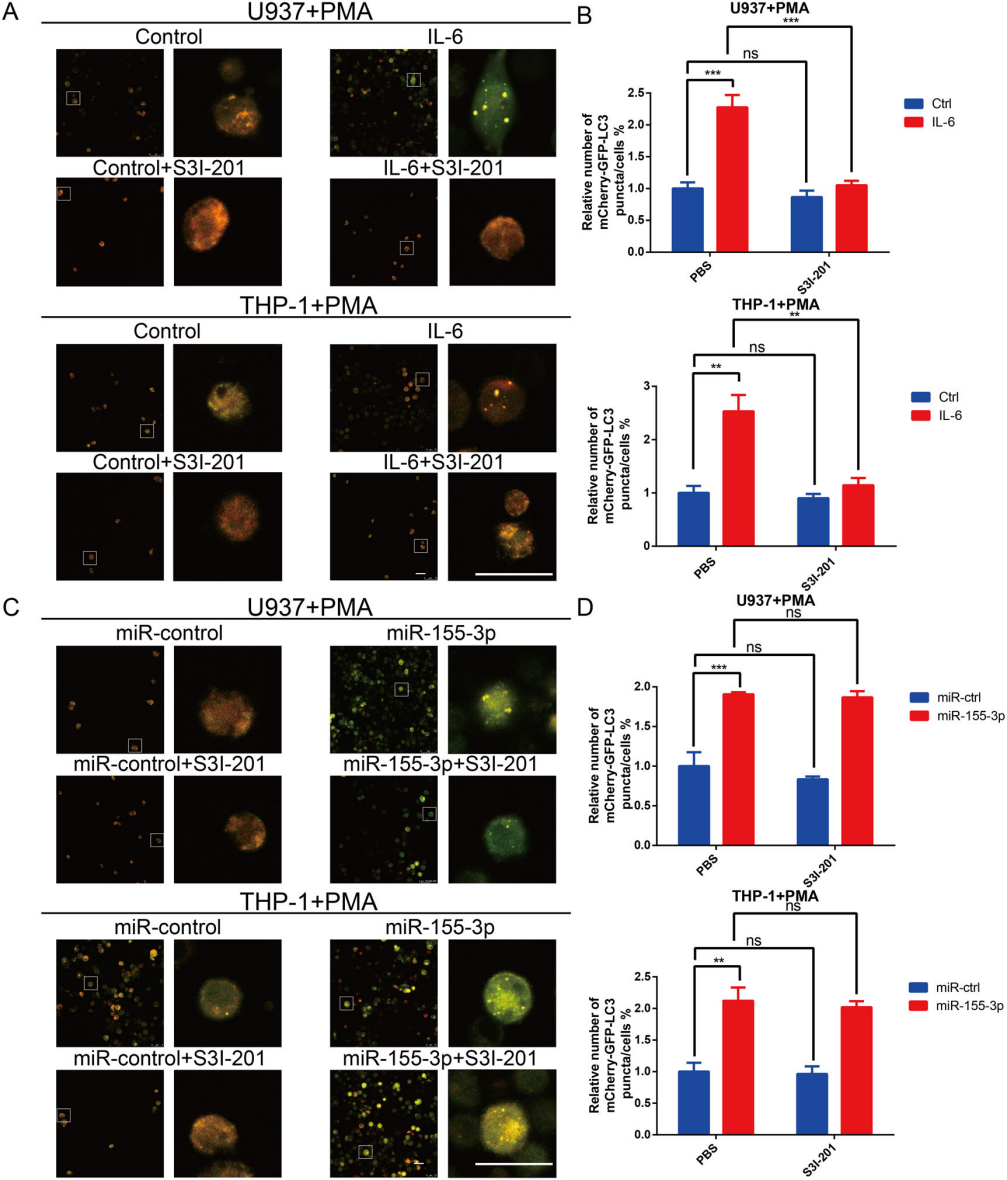
IL-6 and miR-155-3p significantly induce autophagy in macrophages in vitro. **A** Human monocyte cell lines U937 and THP-1 were transfected with lentiviruses expressing mCherry‐GFP‐LC3 plasmid. Then, the mCherry‐GFP‐LC3-U937 and mCherry‐GFP‐LC3-THP-1 cells were transfected with control or IL-6 and incubated with PMA (100 ng/ml) for 24 h in vitro to induce them to differentiate into macrophages. S3I-201 was used to inhibit STAT3 activation. Macrophages of different groups were visualized by confocal microscopy. Representative images are shown (Scale bar, 25 μm). **B** The number of mCherry‐GFP-LC3 puncta in each cell was quantified. **C** The mCherry‐GFP‐LC3-U937 and mCherry‐GFP‐LC3-THP-1 cells were transfected with miR-control or miR-155-3p and incubated with PMA (100 ng/ml) for 24 h in vitro to induce them to differentiate into macrophages. S3I-201 was used to inhibit STAT3 activation. Macrophages of different groups were visualized by confocal microscopy. Representative images are shown (Scale bar, 25 μm). D The number of mCherry‐GFP-LC3 puncta in each cell was quantified. (*P < 0.05; **P < 0.01; ***P < 0.001; *n* = 3).

Next, we examined whether IL-6 could independently induce autophagy in macrophages. Exogenous IL-6 significantly increased the puncta/cell ratio in macrophages (Fig. 5A, B). Studies have increasingly shown that IL-6 triggers autophagy by activating the STAT3 pathway^31,38,39^. Therefore, we used S3I-201, a STAT3 inhibitor, to attenuate IL-6 overexpression. The data showed that the blockade of IL-6 repressed autophagy in macrophages (Fig. 5A, B). Furthermore, western blot analysis showed that IL-6 activated the STAT3 pathway and induced autophagy, which was inhibited by S3I-201 (Fig. S3a). These results revealed that IL-6 triggers autophagy via STAT3 activation in macrophages.

### MiR-155-3p is involved in IL-6-induced autophagy by directly targeting CREBRF in macrophages

Using JASPAR (http://jaspar.genereg.net), we identified many binding sites of STAT3 in the promoter region of MIR155HG, the host gene of miR-155-3p, suggesting that p-STAT3 (Y705) could transcriptionally increase miR-155-3p expression. To investigate the association of IL-6 and miR-155-3p, qRT-PCR was performed. The qRT-PCR results showed that miR-155-3p expression in macrophages overexpressing IL-6 was increased, and S3I-201 eliminated the promoting effect of the IL-6-pSTAT3 pathway on miR-155-3p expression (Fig. S3b). Our team previously reported that miR-155-3p enhances hypoxia-induced autophagy^31^. Interestingly, the overexpression of miR-155-3p promoted autophagy in macrophages, which could not be blocked by S3I-201 (Fig. 5C, D); these findings were confirmed by western blot analysis (Fig. S3c). Furthermore, we found that overexpression of miR-155-3p activated the STAT3 pathway (Fig. S3c). Because autophagy induces STAT3 pathway activation, we inhibited autophagy and found that STAT3 phosphorylation was restrained (Fig. S3d)^32,33^. Next, we searched for potential miR-155-3p target genes in the online miRNA target prediction websites miRDB (http://mirdb.org/miRDB/index.html) and TargetScan (http://www.targetscan.org). According to the common results of these two miR target prediction websites, we identified CREB3 regulatory factor (CREBRF) as highly likely to be the potential target gene of miR-155-3p. CREBRF, a negative regulator of CREB3, is a suppressor of autophagy via the CREB3-ATG5 pathway^40^. To further assess the direct inhibitory effect of miR-155-3p on CREBRF gene transcription, we performed luciferase reporter assays. A vector encoding the wild-type (WT) sequence of the 3′ untranslated region (3′ UTR) of CREBRF mRNA or a vector encoding the mutant (MUT) sequence of the 3’ UTR of CREBRF mRNA lacking the predicted miR-155-3p target site was transfected into macrophages. The putative target site of miR-155-3p in the 3′ UTR of CREBRF is illustrated in Fig. S4a. The relative luciferase activity of the WT construct of the CREBRF 3′ UTR in miR-155-3p-transfected cells decreased to approximately 40% compared with that of the control miRNA, but the MUT construct of the CREBRF 3′ UTR abolished the suppressive effect of miR-155-3p (Fig. S4b). Interestingly, qRT-PCR and western blot analyses demonstrated that the overexpression of miR-155-3p induced a strong decrease in CREBRF expression in macrophages (Fig. S4c, d). The above results showed that miR-155-3p promotes autophagy by directly targeting CREBRF in macrophages.

### IL-6- and miR-155-3p-induced autophagy promotes M2-like macrophage polarization via STAT3 activation

**Fig. 6:**
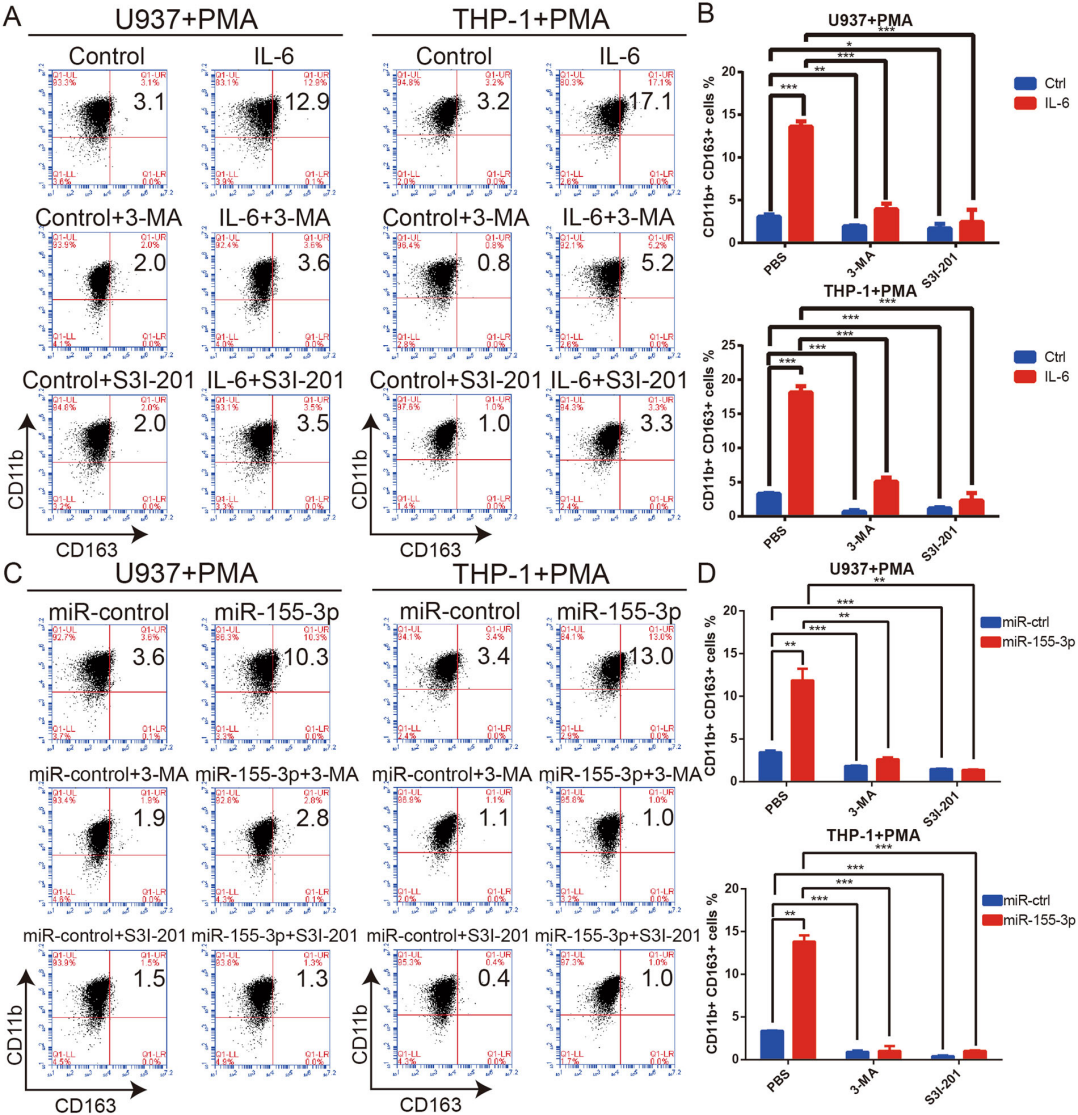
IL-6 and miR-155-3p-induced autophagy promote M2-like macrophage polarization via STAT3 activation. **A**, **B** Human monocyte cell lines U937 and THP-1 were transfected with control or IL-6 and incubated with PMA (100 ng/ml) for 24 h in vitro to induce them to differentiate into macrophages. 3-MA was used to inhibit autophagy. S3I-201 was used to inhibit STAT3 activation. Flow cytometry and quantification were performed to analyze the proportion of CD11b + CD163 + macrophages. **C**, **D** Human monocyte cell lines U937 and THP-1 were transfected with miR-control or miR-155-3p and incubated with PMA (100 ng/ml) for 24 h in vitro to induce them to differentiate into macrophages. 3-MA was used to inhibit autophagy. S3I-201 was used to inhibit STAT3 activation. Flow cytometry and quantification were performed to analyze the proportion of CD11b + CD163 + macrophages. (*P < 0.05; **P < 0.01; ***P < 0.001; *n* = 3).

Autophagy enhances M2-like macrophage polarization by activating the STAT3 pathway^32,33^. The qRT-PCR results showed that the overexpression of IL-6 or miR-155-3p increased the expression of CD163 and decreased the expression of TNFA (Fig. S5a). Moreover, the flow cytometry results showed that IL-6 or miR-155-3p overexpression significantly increased CD163 expression (Fig. 6A–D). The ELISA results showed that IL-6 and miR-155-3p could significantly increase the secretion of IL-10 in macrophages (Fig. S5b). To further investigate whether IL-6 and miR-155-3p promote M2-like macrophage polarization by autophagy activation, 3-MA was used to inhibit autophagy induction. We found that the effect of miR-155-3p was completely blocked; however, the effect of IL-6 was incompletely attenuated (Fig. 6A–D, Fig. S5a, b). Considering that STAT3 pathway activation promotes M2-like macrophage polarization, we suggest that IL-6-induced autophagy strengthens the effect of the IL-6-pSTAT3 pathway in macrophages^41,42^. To test the relationship between the STAT3 pathway and M2-like macrophage polarization, the STAT3 pathway was inhibited by S3I-201. The increased CD163 expression, decreased TNFA expression, and increased secretion of IL-10 were entirely inhibited, which demonstrated that IL-6 and miR-155-3p promote M2-like macrophage polarization via STAT3 activation (Fig. 6A–D, Fig. S5b). Collectively, these results suggested that the IL-6-pSTAT3-miR-155-3p-autophagy-pSTAT3 positive feedback loop promoted M2-like macrophage polarization.

### Overexpression of IL-6 and miR-155-3p in macrophages promotes glioma tumorigenesis in vitro and in vivo

**Fig. 7:**
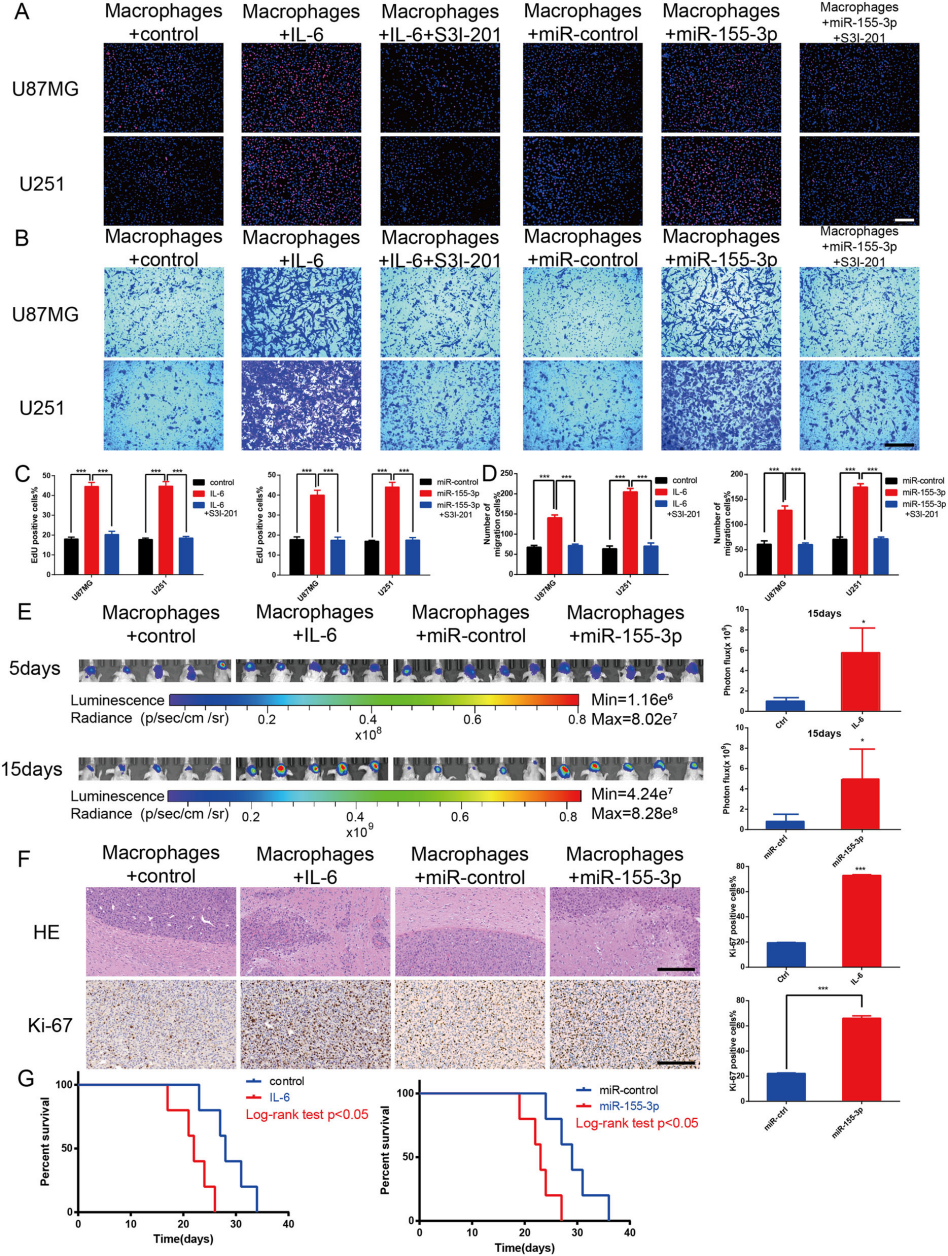
Macrophage overexpression of IL-6 or miR-155-3p promotes glioma progression in vitro and vivo. **A**, **C** An EdU assay evaluated the proliferation of U87MG or U251 cells cocultured with macrophages transfected with control, IL-6, miR-control, or miR-155-3p. S3I-201 was used to inhibit STAT3 activation. The results were quantified (scale bar, 100 μm). **B**, **D** The migration capacity of U87MG or U251 cells cocultured with conditioned macrophages was determined. Representative images of migratory cells and quantifications are shown (scale bar, 200 μm). **E** In vivo bioluminescent imaging analysis of tumor growth in xenograft nude mice bearing U87MG cells with control-macrophages, IL-6-macrophages, miR-control-macrophages or miR-155-3p-macrophages. Representative images on day 5 and 15 post-implantation are shown (data are from five mice per group). **F** HE staining and IHC staining for Ki-67 of sections from xenograft mouse brains with U87MG and control-macrophages, U87MG and IL-6-macrophages, U87MG and miR-control-macrophages or U87MG and miR-155-3p-macrophages on the day of euthanasia (scale bar, 200 μm). **G** Survival analysis of animals implanted with U87MG and control-macrophages, U87MG and IL-6-macrophages, U87MG and miR-control-macrophages or U87MG and miR-155-3p-macrophages (*P* < 0.05 by log-rank analysis; data from five animals per group). (*P < 0.05; **P < 0.01; ***P < 0.001).

To validate the function of IL-6 and miR-155-3p in vitro, U937 cells were transfected with lenti-ov-IL-6, lenti-control, lenti-ov-miR-155-3p or lenti-miR-control virus and incubated with PMA (100 ng/ml) for 24 h in vitro to induce them to differentiate into macrophages. EdU and transwell assays were performed. The results showed that macrophages overexpressing IL-6 or miR-155-3p significantly promoted glioma cell proliferation and migration, which were inhibited by S3I-201 (Fig. 7A–D). Moreover, the macrophages and glioma cells were co-implanted into nude mice to establish orthotopic xenografts, which were used to investigate the effects of IL-6 and miR-155-3p on macrophages. In vivo bioluminescent imaging showed that nude mice implanted with glioma cells and IL-6- or miR-155-3p-overexpressing macrophages had stronger bioluminescence signals than those in the control group (Fig. 7E). Moreover, HE and IHC staining showed that the IL-6 or miR-155-3p-overexpressing macrophage group had tumors with a more obscure border and higher proliferative capacity (Fig. 7F). In addition, the co-implantation of glioma cells and IL-6 or miR-155-3p-overexpressing macrophages resulted in a worse survival time (Fig. 7G). Therefore, the results demonstrated that macrophages overexpressing IL-6 or miR-155-3p promote glioma progression in vivo.

## Discussion

Glioma is the most common malignant tumor in the central nervous system^43^. Immunotherapy for glioma has made substantial progress as a new treatment strategy, but few clinical trials have been effective^44–47^. The main challenge of glioma immunotherapy is the immunosuppressive glioma microenvironment^48,49^. Gliomas change the phenotypes of normal human immune cells to promote progression^50,51^. Many studies have demonstrated that TAMs are the main infiltrating immune cells in the glioma microenvironment and promote glioma progression^52–55^. Therefore, elucidating the mechanism of TAMs in the formation of the immunosuppressive glioma microenvironment will provide an important reference for improving glioma immunotherapy. Emerging evidence has confirmed that exosomes participate in intercellular communication by delivering their contents to recipient cells^16^. Hypoxia is one of the significant hallmarks of the glioma microenvironment and is involved in glioma progression in the immunosuppressive tumor microenvironment^56–58^. Our previous study found that H-GDEs could promote the immunosuppressive function of cells in the glioma microenvironment^14,22,23^. However, the mechanism by which TAMs develop increased immunosuppressive function is unknown. Autophagy plays a significant role in promoting M2-like macrophage polarization via activation of the STAT3 pathway^32,33^. Therefore, we investigated the effect and mechanism of H-GDEs on autophagy and M2-like macrophage polarization. We found that H-GDEs could induce autophagy and promote M2-like macrophage polarization, suggesting that autophagy may strengthen the immunosuppressive function of TAMs caused by H-GDEs. Next, we found that an inhibitor of autophagy, 3-MA, could also inhibit M2-like macrophage polarization caused by H-GDEs, indicating that autophagy promotes M2-like macrophage polarization.

Tumor-derived exosomes have been reported to deliver proteins and genetic materials to transform the phenotype of recipient immune cells in both local and remote areas^59,60^. Previous studies by our group determined that IL-6 and miR-155-3p are overexpressed and promote autophagy in glioma^31^. IL-6 is associated with autoimmune diseases, cancer, obesity, diabetes, depression and anxiety; currently, many articles regarding IL-6 are being published, continuing to clarify its key role in different biological processes and indicating the important role of this cytokine^61,62^. Lamano et al.^63^ reported that IL-6 induces peripheral myeloid PD-L1 in GBM. However, no articles have previously reported that IL-6 is delivered to TAMs by H-GDEs and promotes M2-like macrophage polarization. In addition, we reported that hypoxia treatment induced a significant increase in IL-6 secretion in the glioma cell culture supernatants, but we did not investigate the effects of secreted IL-6 on the immunosuppressive tumor microenvironment. In this way, we suggest that IL-6 might be delivered by exosomes, small membrane vesicles secreted by several cell types^15^. Meanwhile, exosomes play an important role in intercellular communication by delivering their contents^16,17^. Tumor-derived exosomes have been shown to suppress the functions of immune cells by transferring genetic material^18^. Interestingly, our current study found that IL-6 expression was significantly increased in H-GDEs. In addition, we discovered that the expression of miR-155-3p, an important component of the pathway, was increased in H-GDEs. Moreover, macrophages treated with H-GDEs had a higher expression level of IL-6 and miR-155-3p than those treated with PBS or N-GDEs, and IL-6 increased the expression of miR-155-3p in TAMs. In this way, H-GDEs may strongly influence TAMs by transmitting the IL-6-miR-155-3p pathway holistically. IL-6 and miR-155-3p promote tumorigenesis in glioma cells, but their functions in TAMs are still not clear^30,64^. It was recently reported that IL-6 is crucial for orchestrating premetastatic niche formation and immunosuppression^65^. In addition, miR-155-3p is associated with immune infiltration and immune checkpoint molecule expression^66^. Afterwards, we detected the functions of IL-6 and miR-155-3p on TAMs. The results showed that IL-6 and miR-155-3p could promote autophagy in TAMs, which indicated that IL-6 and miR-155-3p delivered by exosomes promote autophagy in TAMs in addition to glioma cells. In addition, we indicated that IL-6 and miR-155-3p could promote M2-like macrophage polarization. Furthermore, we confirmed that the overexpression of IL-6 and miR-155-3p in TAMs promotes glioma progression in vitro and in vivo.

The positive feedback loop plays a significant role in driving tumorigenesis^38,67^. The STAT3 pathway is involved in the progression of various tumors^68^. Many studies have reported that the IL-6-pSTAT3 pathway induces autophagy^31,38,39^. In addition, we found that IL-6 increased miR-155-3p expression by STAT3 activation in TAMs. Moreover, autophagy has been reported to induce STAT3 pathway activation^38^. Activation of the STAT3 pathway is involved in M2-like macrophage polarization^41,69^. S3I-201, a STAT3 inhibitor, was used to test the role of pSTAT3 in the pathways of IL-6- and miR-155-3p-induced M2-like macrophage polarization. We found that IL-6-induced autophagy was inhibited by S3I-201, whereas miR-155-3p-induced autophagy was not inhibited by S3I-201. Therefore, we propose that miR-155-3p can promote autophagy via a pathway other than STAT3. Next, we found that miR-155-3p served as a competing endogenous RNA (ceRNA) to target CREBRF, a suppressor of autophagy in macrophages^40^. Thus, overexpression of miR-155-3p might rescue the function of S3I-201 which inhibited the IL-6-pSTAT3 pathway-induced autophagy. To investigate the role of autophagy in this pathway, we inhibited autophagy using 3-MA. 3-MA inhibited miR-155-3p-induced M2-like polarization entirely, which demonstrated that miR-155-3p promotes M2-like polarization via the autophagy-pSTAT3 pathway. However, the influence of IL-6 on M2-like macrophage polarization was not completely inhibited, which suggested that IL-6 increases the level of M2-like macrophage polarization directly via the IL-6-pSTAT3 pathway and indirectly via the IL-6-autophagy-pSTAT3 pathway. The autophagy-pSTAT3 pathway increased the effect of IL-6 on M2-like macrophage polarization. To examine the significant role of the STAT3 pathway in M2-like macrophage polarization, we inhibited the STAT3 pathway. We found that M2-like macrophage polarization was inhibited after inhibiting the STAT3 pathway. Overall, there may be an IL-6-pSTAT3-miR-155-3p-autophagy-pSTAT3 positive feedback loop to promote M2-like macrophage polarization (Fig. S6). In addition, we previously reported that TAM-derived exosomes promote glioma progression^70^. Interestingly, our current study found that GDEs strengthened TAM-mediated immunosuppression. In this way, we found a similar interrelationship between glioma cells and TAMs: glioma cells promote M2-like macrophage polarization and TAMs accelerate glioma progression, which is a similar positive feedback loop. Exosomes, reported to be important in tumor progression, play a key role in this positive feedback loop^71^.

Our studies aimed to determine the mechanism of H-GDE-induced immunosuppressive function of TAMs. In addition, we found that IL-6 and miR-155-3p play an important role in M2-like macrophage polarization. Moreover, the IL-6-pSTAT3-miR-155-3p-autophagy-pSTAT3 positive feedback loop strengthened this effect.

In summary, we demonstrated that H-GDE-derived IL-6 and miR-155-3p can induce M2-like macrophage polarization via the IL-6-pSTAT3-miR-155-3p-autophagy-pSTAT3 positive feedback loop, which promotes glioma progression. Our findings also show that IL-6 and miR-155-3p may be novel biomarkers for diagnosing glioma and that treatment targeting autophagy and the STAT3 pathway may impair the immunosuppressive tumor microenvironment and participate in antitumor immunotherapy.

## Supplementary information

Supplementary Figure S1

Supplementary Figure S2

Supplementary Figure S3

Supplementary Figure S4

Supplementary Figure S5

Supplementary Figure S6

Supplementary Figure Legends
