# Supplementary figures and images for: Hypoxic glioma-derived exosomes promote M2-like macrophage polarization by enhancing autophagy induction
Source: Cell Death Dis. 2021 Apr 7;12(4):373. doi: 10.1038/s41419-021-03664-1 (PMC8026615; doi:10.1038/s41419-021-03664-1)

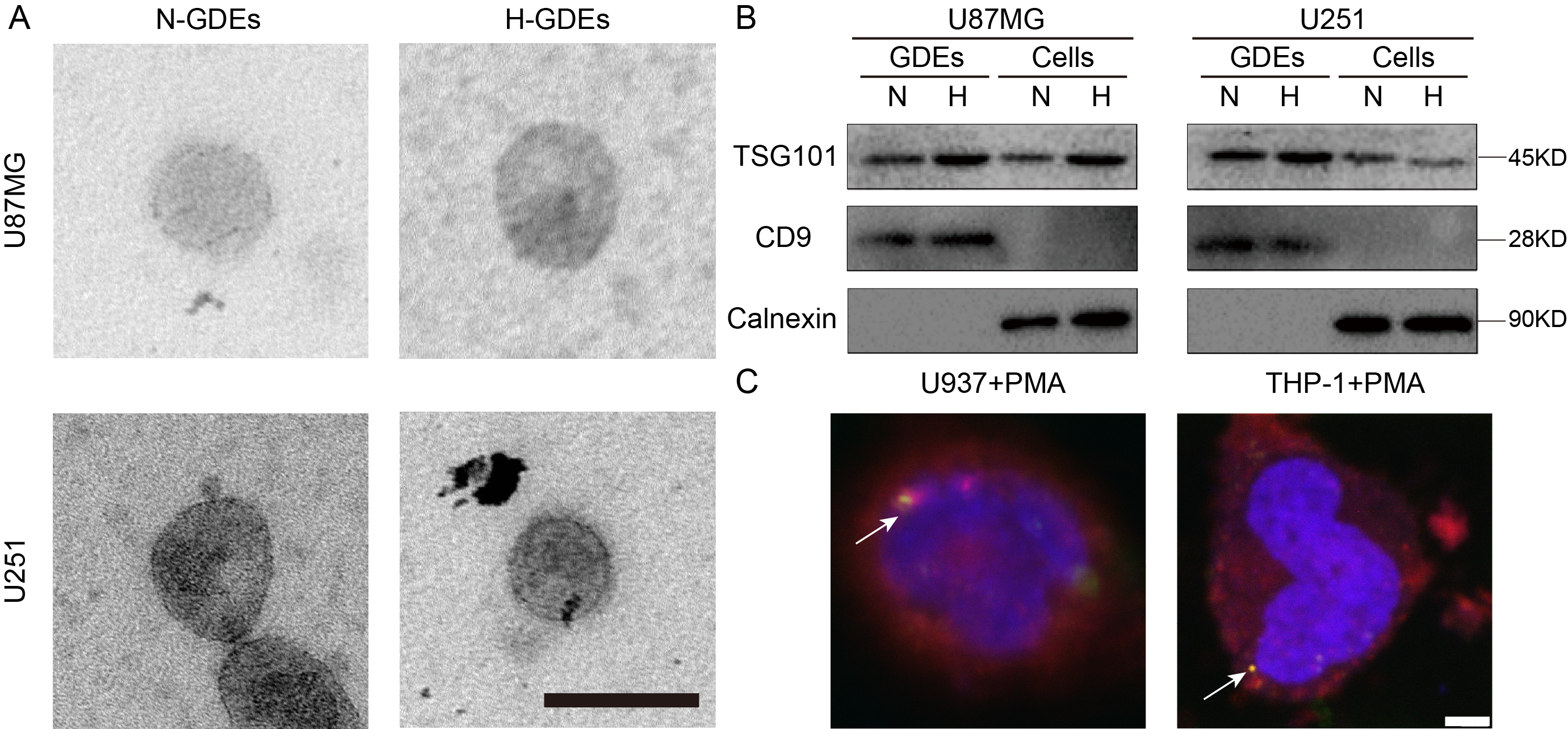

Supplement: Supplementary file 1 — Supplementary Figure S1 [file 41419_2021_3664_MOESM1_ESM.tif]

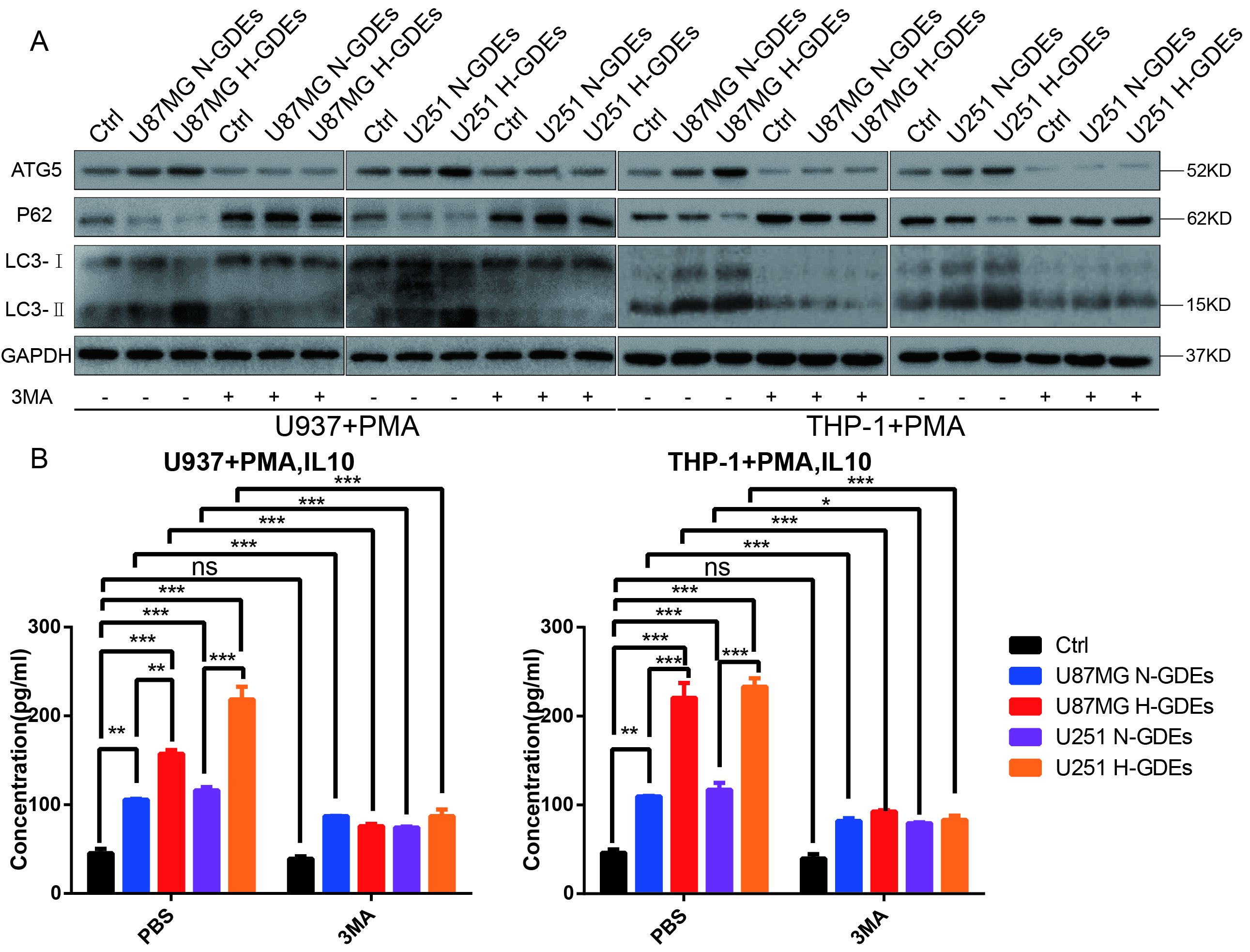

Supplement: Supplementary file 2 — Supplementary Figure S2 [file 41419_2021_3664_MOESM2_ESM.tif]

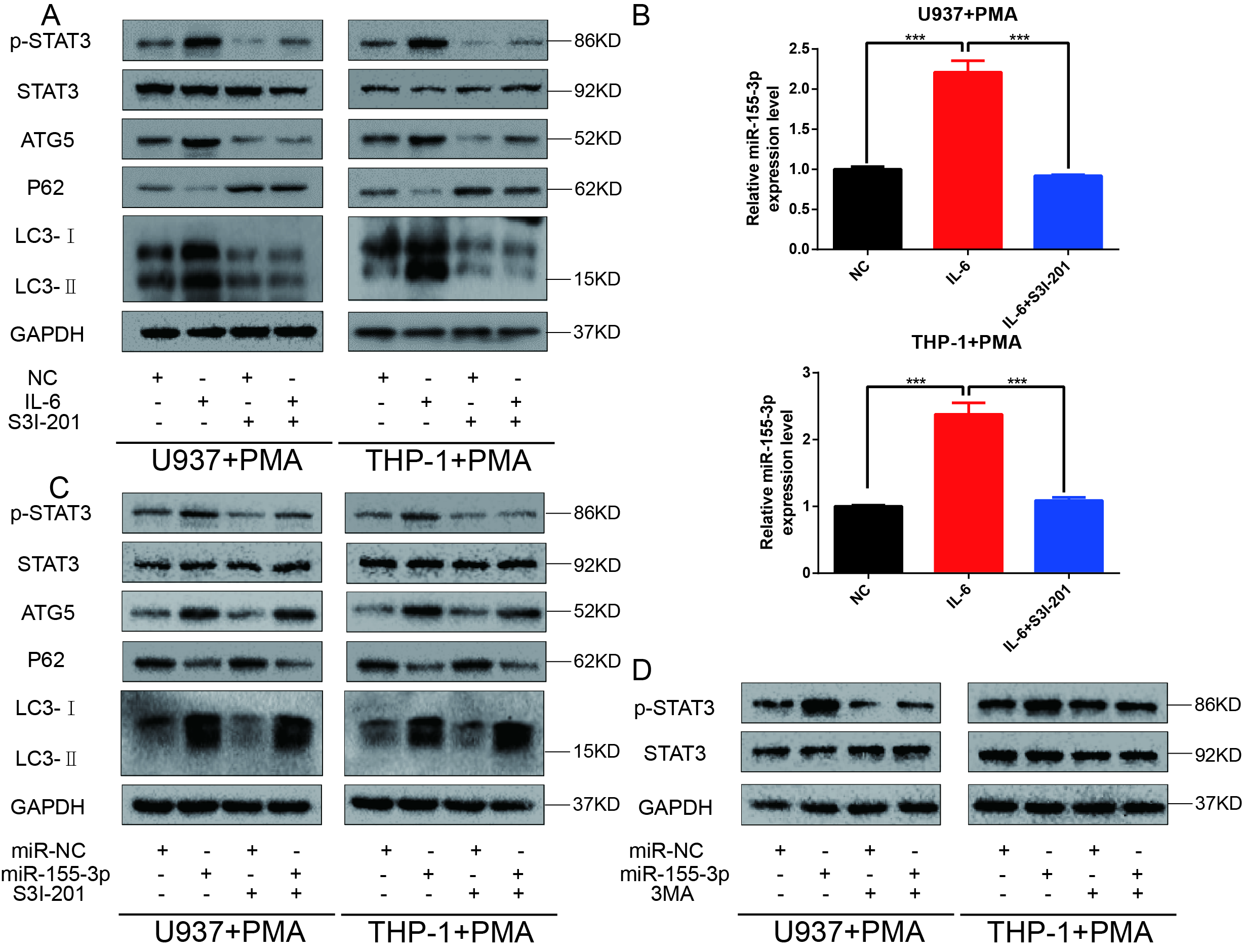

Supplement: Supplementary file 3 — Supplementary Figure S3 [file 41419_2021_3664_MOESM3_ESM.tif]

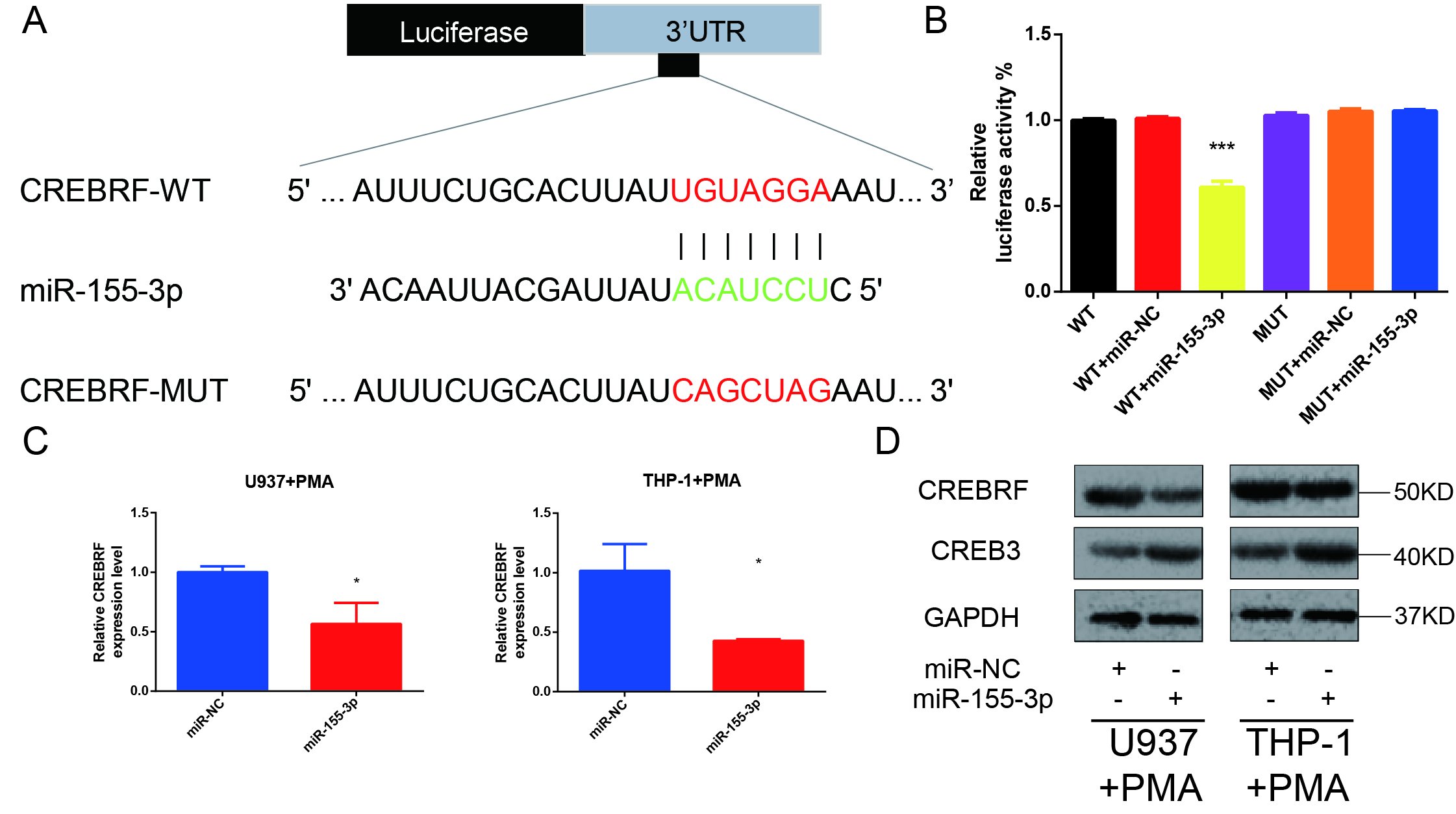

Supplement: Supplementary file 4 — Supplementary Figure S4 [file 41419_2021_3664_MOESM4_ESM.tif]

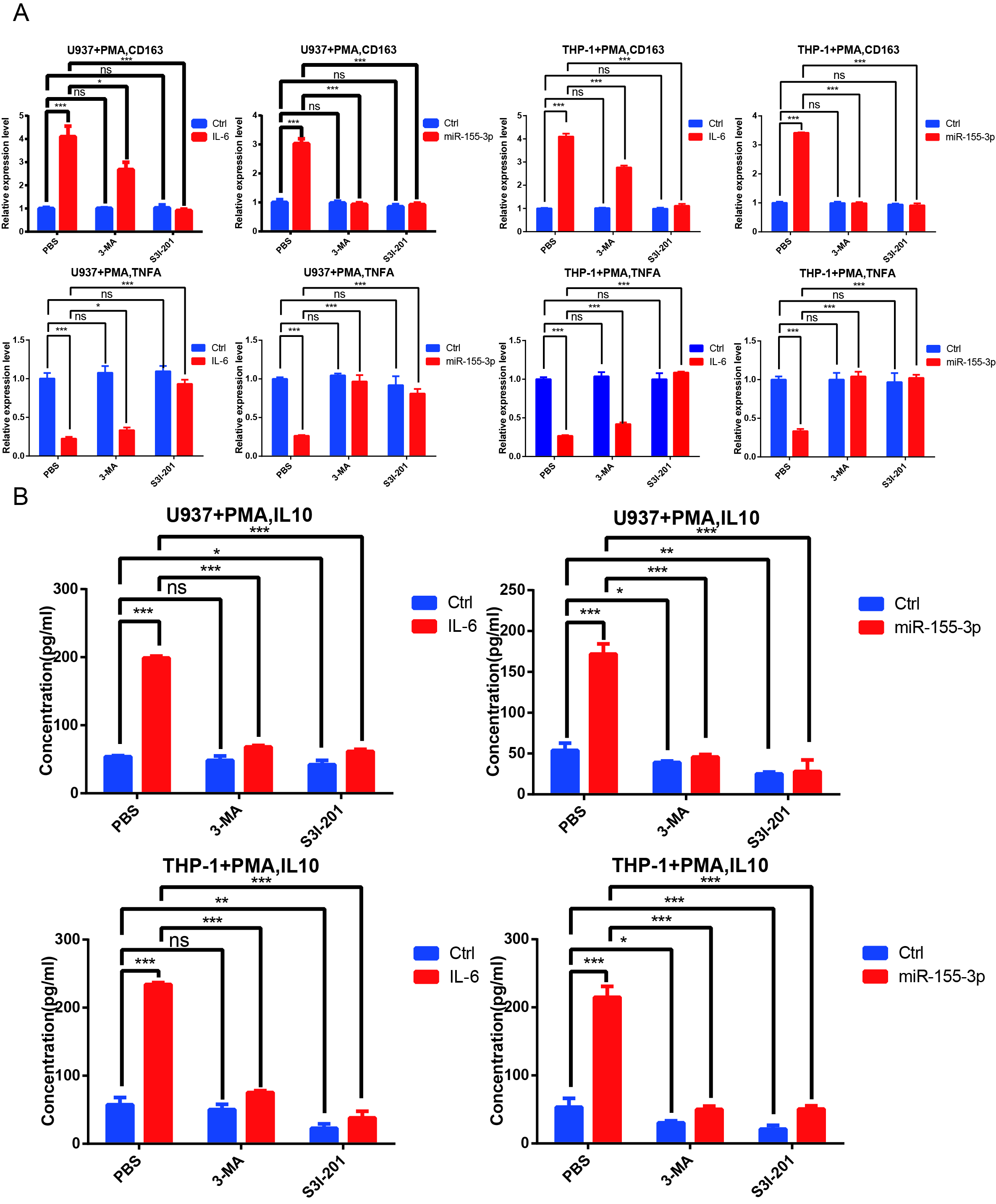

Supplement: Supplementary file 5 — Supplementary Figure S5 [file 41419_2021_3664_MOESM5_ESM.tif]

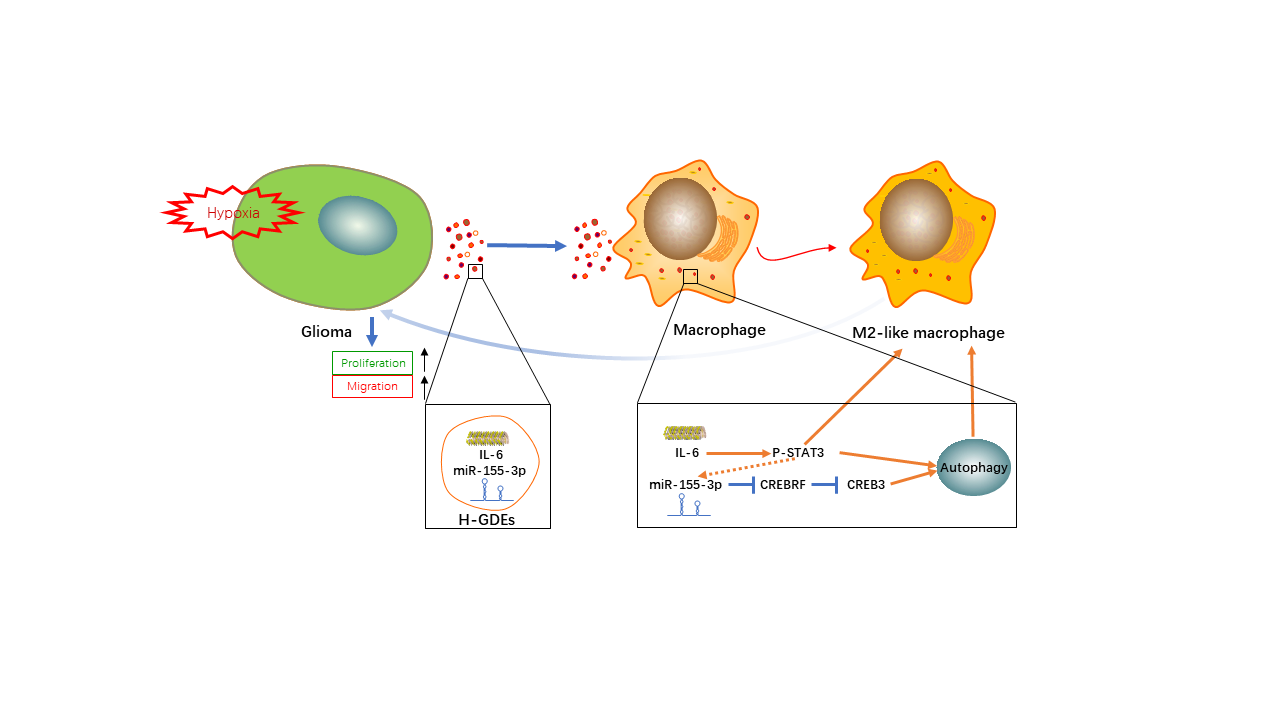

Supplement: Supplementary file 6 — Supplementary Figure S6 [file 41419_2021_3664_MOESM6_ESM.tif]
