# Supplementary material for: Hypoxic glioma-derived exosomes promote M2-like macrophage polarization by enhancing autophagy induction
Source: Cell Death Dis. 2021 Apr 7;12(4):373. doi: 10.1038/s41419-021-03664-1 (PMC8026615; doi:10.1038/s41419-021-03664-1)
Supplement: Supplementary file 7 — Supplementary Figure Legends [file 41419_2021_3664_MOESM7_ESM.docx]

**Supplementary Figure Legends**

**Figure S1. Identification of GDEs and internalization of H-GDEs by macrophages.**

(A) Representative electron micrograph of exosomes isolated from normoxic and hypoxic U87MG and U251 conditioned medium. Scale bar, 100 nm. (B) Western blot analysis of the exosome-positive marker TSG101, CD9 and the exosome-negative marker calnexin in N-GDEs, H-GDEs and glioma cells. (C) Representative confocal microscopy images showing the internalization of PKH67-labeled H-GDEs (green) by macrophages. Scale bar, 4 μm.

**Figure S2. H-GDEs significantly induce autophagy and M2-like polarization in macrophages in vitro.**

(A) The macrophages were treated with PBS, N-GDEs or H-GDEs isolated from the culture supernatants of U87MG or U251 cells. 3-MA was used to inhibit autophagy. ATG5, P62 and LC3B levels were examined by western blot analysis. GAPDH was used as a loading control. (B) H-GDEs increased the secretion of IL10 in macrophage culture supernatants, as revealed by ELISA (*P < 0.05; **P < 0.01; ***P < 0.001; n = 3).

**Figure S3. IL-6 and miR-155-3p promote autophagy and STAT3 activation in macrophages.**

(A, B) Human monocyte cell lines U937 and THP-1 were transfected with control or IL-6 and incubated with PMA (100 ng/ml) for 24 h in vitro to induce them to differentiate into macrophages. S3I-201 was used to inhibit STAT3 activation. P-STAT3, STAT3, ATG5, P62 and LC3B levels were examined by western blot analysis. MiR-155-3p levels were examined by qRT-PCR. GAPDH was used as a loading control. (C) Human monocyte cell lines U937 and THP-1 were transfected with miR-control or miR-155-3p and incubated with PMA (100 ng/ml) for 24 h in vitro to induce them to differentiate into macrophages. S3I-201 was used to inhibit STAT3 activation. P-STAT3, STAT3, ATG5, P62 and LC3B levels were examined by western blot analysis. GAPDH was used as a loading control. (D) Human monocyte cell lines U937 and THP-1 were transfected with miR-control or miR-155-3p and incubated with PMA (100 ng/ml) for 24 h in vitro to induce them to differentiate into macrophages. 3-MA was used to inhibit autophagy. P-STAT3 and STAT3 levels were examined by western blot analysis. GAPDH was used as a loading control (*P < 0.05; **P < 0.01; ***P < 0.001; n = 3).

**Figure S4. CREBRF is a direct target of miR-155-3p in macrophages.**

(A) miR-155-3p and its putative binding sequence in the wild-type (WT) and mutant (MUT) 3’-UTR of CREBRF. (B) Overexpression of miR-155-3p significantly decreased the luciferase activity that carried wild-type (WT) but not mutant type (MUT) 3’-UTR of CREBRF. (C, D) Overexpression of miR-155-3p markedly suppressed the mRNA and protein levels of CREBRF in macrophages. GAPDH was used as a loading control (*P < 0.05; **P < 0.01; ***P < 0.001; n = 3).

**Figure S5. IL-6 and miR-155-3p promote M2-like macrophage polarization.**

(A) Human monocyte cell lines U937 and THP-1 were transfected with control, IL-6, miR-control, or miR-155-3p and incubated with PMA (100 ng/ml) for 24 h in vitro to induce them to differentiate into macrophages. S3I-201 was used to inhibit STAT3 activation. 3-MA was used to inhibit autophagy. The expression levels of CD163 and TNFA were determined by qRT-PCR. (B) Overexpression of IL-6 or miR-155-3p increased the secretion of IL10 in macrophage culture supernatants, as revealed by ELISA (*P < 0.05; **P < 0.01; ***P < 0.001; n = 3).

**Figure S6. Schematic diagram.**
